# Hospital clones of methicillin-resistant *Staphylococcus aureus* are carried by medical students even before healthcare exposure

**DOI:** 10.1186/s13756-017-0175-2

**Published:** 2017-01-23

**Authors:** Ido Orlin, Assaf Rokney, Avi Onn, Daniel Glikman, Avi Peretz

**Affiliations:** 1 The Faculty of Medicine in the Galilee, Bar-Ilan University, Galilee, Israel; 2 National Staphylococcus aureus Reference Center, Central Laboratories, Israel Ministry of Health, Jerusalem, Israel; 3 Pediatric Gastrointestinal Unit, Padeh Poriya Medical Center, Poriya, Tiberias Israel; 4 Pediatric Infectious Diseases Service, Galilee Medical Center, Nahariya, Israel; 5 Clinical Microbiology Laboratory, Padeh Poriya Medical Center, Poriya, Hanna Senesh 818/2, Tiberias, Israel

**Keywords:** Methicillin-resistance, Review, *Staphylococcus aureus*, Carriage, Medical students, Community-associated, Healthcare-associated

## Abstract

**Background:**

Methicillin-resistant *Staphylococcus aureus* (MRSA) strains are prevalent in healthcare and the community. Few studies have examined MRSA carriage among medical students.

The aim of this study is to examine *Staphylococcus aureus* (SA) carriage, and particular MRSA, over time in cohort medical students

**Methods:**

Prospective collection of nasal swabs from medical students in Israel and assessment of SA carriage. Three samples were taken per student in preclinical and clinical parts of studies. Antibiotic susceptibilities were recorded and MRSA typing was performed by staphylococcal cassette chromosome *mec* (SCC*mec*) types, Panton Valentine Leukocidin (PVL) encoding genes, and *spa* types. Clonality was assessed by pulsed-field gel electrophoresis.

**Results:**

Among 58 students, SA carriage rates increased from 33% to 38% to 41% at baseline (preclinical studies), 13 and 19 months (clinical studies), respectively (*p* = 0.07). Methicillin-susceptible SA (MSSA) carriage increased in the clinical studies period (22 to 41%, *p* = 0.01). Overall, seven students (12%) carried 13 MRSA isolates. MRSA isolates were PVL negative and were characterized as SCC*mec*II-t002, SCC*mec*IV-t032, or t12435 with untypable SCC*mec*. MRSA carriage during the pre-clinical studies was evident in 4/7 students. Two students carried different MRSA clones at various times and persistent MRSA carriage was noted in one student. Simultaneous carriage of MRSA and MSSA was not detected.

**Conclusions:**

MSSA carriage increased during the clinical part of studies in Israeli medical students. Compared with previous reports, higher rates of MRSA carriage were evident. MRSA strains were genotypically similar to Israeli healthcare-associated clones; however, carriage occurred largely before healthcare exposure, implying community-acquisition of hospital strains.

## Background

*Staphylococcus aureus* (SA) is a versatile pathogen capable of colonizing humans and mammals, and causing skin and invasive diseases such as endocarditis, osteomyelitis, and severe sepsis. Methicillin-resistant SA (MRSA) was reported in 1961, soon after the introduction of the semisynthetic penicillin, methicillin [1]. Until the 1990s, MRSA strains were associated mostly with hospitals and infected patients who had contact with the healthcare system, hence the name healthcare-associated MRSA (HA-MRSA). These strains were typically resistant to multiple antimicrobials (multi-drug resistant – MDR) and carried large staphylococcal cassette chromosome *mec* (SCC*mec*) elements, mostly types I-III [2]. Beginning in the late 1990s, community-associated strains of MRSA (CA-MRSA) were described with increasing incidence, colonizing and infecting patients who had no recent contact with the healthcare system. These strains were usually non-MDR, carried small SCC*mec* elements, mostly types IV and V, and also carried the genes encoding the Panton-Valentine leukocidin (PVL), a pore-forming toxin rarely found among SA isolates [1]. Nowadays, in many geographic regions there are no longer lineages, genetic markers, or susceptibility phenotypes that are indicative for specific epidemiological origins such as HA-MRSA or CA-MRSA [1].

The primary reservoir of SA is the anterior nares, but the organism can be isolated from multiple sites. The prevalence and incidence of SA nasal carriage vary according to the population studied. According to multiple cross-sectional surveys, the mean carriage rate across the general population is 37% [3]. Regarding MRSA carriage in the era of CA-MRSA, the overall MRSA carriage rate in the United States has increased from 0.8% in 2001–2002 to 1.5% in 2003–2004, and it is particularly important in the hospital environment because colonized and infected patients represent the most important reservoir of MRSA in healthcare facilities [4, 5].

The carriage of MRSA among healthcare workers (HCW) due to the relation of their exposure to patients harboring these strains is a well-known phenomenon [6]. Medical students seem to be no exception, as their clinical study environment involves various medical settings such as hospitals and community clinics, which may contribute to their carriage status. In a non-systematic review of the English literature we found several studies, conducted worldwide, investigating SA carriage among medical students (Table 1) [7–32]. These studies reported MRSA carriage rates of 0–5.4% and SA carriage rates of 14–45% (excluding a small study from India reporting a higher carriage rate and a study from Canada of dental students [12, 15]). Only in a minority of these studies assessed the difference of SA carriage between pre-clinical and clinical medical students and most investigations assessed carriage at a single time-point only [7, 9, 21–23, 26, 27, 31]. Of these, three studies demonstrated higher SA carriage rates in the clinical studies stage [9, 23, 26]. Furthermore, only a single study from Thailand had prospectively examined the change in SA carriage in the same group of medical students over multiple sampling times [26]. Therefore, we aimed to prospectively examine the change in SA carriage among medical students transitioning from pre-clinical to clinical education with a focus on MRSA. Moreover, we aimed to characterize MRSA isolates from medical students using SCC*mec* and *spa* typing as well as PFGE.

**Table 1:** Staphylococcus aureus carriage among medical students in published papers

Year	Reference	Country	Population	SA Carriage, n (%)			MRSA Carriage, n (%)			Notes
				Total	Pre-clinical group	Clinical group	Total	Pre-clinical group	Clinical group	
1994	Stubbs et al. [7]	Australia	*N* = 808	261/808 (30%)	68/193 (35%)	252/615 (40%)	0	0	0	
2004	Berthelot et al. [8]	France	*N* = 124	34/124 (27%)	N/A	N/A	1/124) (1%)	N\A	N/A	
2007	Güçlü et al. [9]	Turkey	*N* = 179	50/179 (28%)	12/179 (15%)	38/179 (76%)^a^	N/A	N/A	N/A	
2007	Higuchi et al. [10]	Japan	*N* = 98	36/98 (36%)	N/A	N/A	0	0	0	
2007	Adesida et al. [11]	Nigeria	*N* = 182	26/182 (14%)	N/A	N/A	0	0	0	
2008	Baliga et al. [12]	India	*N* = 50	44/50 (88%)	N/A	N/A	12/50 (24%)	N/A	N/A	
2009	Slifka et al. [13]	USA	*N* = 182	62/182 (34%)	N/A	N/A	5/182 (3%)	2/95(2%)	3/87 (3%)	
2011	Ma et al. [14]	China	*N* = 2103^b^	212/2103 (10%)	N/A	N/A	22/2103 (1%)	N/A	N/A	SCC*mec* I, ST88 PVL- (*n* = 1); SCC*mec* II, ST5 PVL+ (*n* = 1) PVL- (*n* = 1); SCC*mec* III, ST 239 PVL- (*n* = 1); SCC*mec* IVa, ST88 PVL+ (*n* = 5), PVL-(*n* = 3), ST30 (*n* = 1), ST1, PVL+ (*n* = 1); SCC*mec* IVn,ST59 (*n* = 2); SCC*mec* IVc, ST30,PVL+ (*n* = 2) SCC*mec* V, ST59 PVL+ (*n* = 2), ST90 PVL- (*n* = 1); non typable (*n* = 1)
2011	Roberts et al. [15]	Canada	*N* = 61^c^	N/A	N/A	N/A	13/61 (21%)	N/A	N/A	SCC*mec* IV, ST1159 (*n* = 1); 12 NT*^,HA^ [ST8,ST30 (*n* = 3), ST39, ST45, ST101, ST109, ST256 (*n* = 2), ST 1474, ST1474]
2011	Chamberlain et al. [16]	USA	*N* = 132	62/132 (47%)	N/A	N/A	7/132 (5%)	N/A	N/A	
2011	Kitti et al. [17]	Thailand	*N* = 200	30/200 (15%)	N/A	N/A	2/200 (1%)	N/A	N/A	SCC*mec* II (*n* = 2)
2012	Gualdoni et al. [18]	Austria	*N* = 79	20/79 (25%)	N/A	N/A	0	N/A	N/A	
2012	Bettin et al. [19]	Colombia	*N* = 387	96/387 (25%)	N/A	N/A	6/387 (2%)	N/A	N/A	SCC*mec* I, PVL- (*n* = 1); SCC*mec* IV, PVL+ (*n* = 5) ^CA^
2012	Mahmutović Vranić et al. [20]	Bosnia	*N* = 387	39/387 (11%)	N/A	N/A	0	N/A	N/A	
2012	Chen et al. [21]	Taiwan	*N* = 322	55/322 (17%)	24/167 (14%)	31/155 (20%)	7/322 (2%)	4/167 (2%)	3/155 (2%)	SCC*mec* IV, ST59 (*n* = 2), N/A MLST (*n* = 4), PVL – (*n* = 6)^CA^ SCC*mec* V_T_, ST59, PVL+ (*n* = 1)^CA^
2012	Syafinaz et al. [22]	Malaysia	*N* = 209	21/209 (10%)	14/111 (13%)	7/97 (7%)	0	0	0	
2013	Bellows et al. [23]	USA	*N* = 94	15/94 (16%)	3/42 (7%)	12/52 (23%)^a^	3/94 (3%)	0	3/52 (6%)	
2013	Cirković et al. [24]	Serbia	*N* = 533	N/A	N/A	N/A	2/533 (0.4%)	N/A	N/A	SCC*mec* IV, ST80^CA^ SCC*mec* V,ST152^CA^
2013	Trépanier et al. [25]	Canada	*N* = 497^d^	N/A	N/A	N/A	1/497 (0.2%)	1/247 (0.4%)	0	
2014	Treesirichod et al. [26]	Thailand	*N* = 128	42/127 (33%)	38/128 (30%)	2^nd^ sample 39/128 (31%) 3^rd^ sample 50/127 (39%)^a^	0	0	0	3 prospective samples
2015	Zakai et al. [27]	Saudi Arabia	*N* = 182	60/182 (32%)	32/32 (100%)	28/150 (19%)	10/182 (5%)	0/32	10/150 (7%)	
2015	Collazos Marín et al. [28]	Colombia	*N* = 216	54 (25%)	N/A	N/A	9/216 (4%)	N/A	N/A	4/9 (44%)^CA^
2015	Ho et al. [29]	China	*N* = 1149	526/1149 (45%)	N/A	N/A	6/1149 (1%)	N/A	N/A	SCC*mec* IV,t437 (n = 3)^CA^ :PVL+ (*n* = 1) t11642 (*n* = 1)^CA^, t1081 (*n* = 1)^HA^, t267 (*n* = 1)^HA^
2016	Baek et al. [30]	South Korea	*N* = 159^c^	N/A	N/A	N/A	5/159 (3%)	N/A		SCC*mec* IV (*n* = 4) SCC*mec* I (n = 1)
2016	Okamo et al. [31]	Tanzania	*N* = 314	66/314 (21%)	33/166 (20%)	33/148 (22%)	1/314 (0.3%)	N/A	N/A	
2016	Ansari et al. [32]	Nepal	*N* = 200	30/200 (15%)	N/A	N/A	8/200 (4%)	N/A	N/A	

*N/A* not available, *CA-MRSA* community-associated methicillin-resistant *Staphylococcus aureus*, *HA-MRSA* hospital-associated methicillin-resistant *Staphylococcus aureus*, *SA Staphylococcus aureus*, SCC*mec* Staphylococcal Cassette Chromosome *mec*

^a^ *p* < 0.05

^b^Medical interns

^c^Dental students

^d^Divided to 250 medical residents and 247 undergrad students including interns

^HA^suggested by article as HA-MRSA

^CA^Suggested by article as CA-MRSA

^*^Not SCC*mec* type I-V

## Methods

### Study population

The study population included medical students at the Faculty of Medicine in the Galilee of Bar-Ilan University, in Safed, Israel. The Faculty has two academic classes: 4-year and 3-year tracks. The 3-year track students finished 3 years of pre-clinical medical education in medical faculties across Europe (such as Hungary, Italy, and Lithuania). Almost all of these students reported no patient encounters during their studies, as most of their education was lecture-based. The 4-year track students have graduated from various pre-med Israeli academic institutions. As no dormitories are available, most students live separately. The study was approved by the local Ethics Committees of Baruch Padeh Medical Center, Poriya, and Bar-Ilan University.

### Sampling

Samples were collected three times for each participant during 2012–2014. Sampling was made by a sterile swab (COPAN, Italy) from the nostrils, independently by the participant. First sampling was performed towards the end of pre-clinical studies, with second and third samplings collected at 13 and 19 months post-initial sampling while students were rotating in clinical clerkships. All samples were kept at room temperature for a maximum of 24 h before being transferred to the microbiology laboratory.

### *Staphylococcus aureu*s identification

Microbiological examinations were performed at the Baruch Padeh Medical Center Microbiology Laboratory in Poriya, Israel. Primary culture was performed by striking technique in order to perform culture and colony isolation on a selective chromogenic growth medium (Chromagar MRSA/MSSA, hylabs, Israel). Confirmatory tests for SA included gram staining, catalase test, and rapid agglutination test for simultaneous detection of the fibrinogen affinity antigen (clumping factor), protein A, and the capsular polysaccharides (Pastorex™ Staph Plus, BIO RAD, France).

### Susceptibility tests

Antimicrobial susceptibility tests were performed by disc diffusion method on Muller-Hinton agar (BD Diagnostics, Sparks, MD) in accordance with the Clinical and Laboratory Standards Institute (CLSI) guidelines for rifampicin, vancomycin, erythromycin, gentamicin, cefotaxime, clindamycin, penicillin, oxacillin, mupirocin, fusidic acid, tetracycline, trimethoprim-sulfamethoxsazole (TMP-SMX), and cefoxitin [33]. MDR for MRSA was defined as resistance to at least three antimicrobial agents in addition to β-lactams.

### Molecular characteristics

Typing of MRSA strains was performed at the National *Staphylococcus aureus* Reference Center, Central Laboratories, Israel Ministry of Health, Jerusalem, Israel.

### PCR detection of *mec*A and PVL genes

PCR reactions in a total volume of 23 mL included 10.5 mL DDW, 12.5 mL PCR mix (ReddyMix, Tamar Laboratory Supplies, Mevaseret Zion), MecA1 and MecA2 primers or Luk-PV-1 and Luk-PV-2 primers (1 mM each). Thermocycling conditions were set at 94 °C for 2 min followed by 30 cycles of 94 °C for 30 s, 55 °C for 30 s, 72 °C for 60 s, followed by a final step of 72 °C for 2 min [34, 35].

### Detection of SCC*mec* types

SCC*mec* types were detected using the method of Zhang et al. [35]. PCR reactions were performed in a total volume of 20 mL, including 9.5 mL DDW, 9.5 mL PCR mix (ReddyMix, Tamar Laboratory Supplies, Mevaseret Zion), and 1 mL primer mix with a final concentration as recommended [35]. Thermocycling conditions were set at 95 °C for 5 min, followed by 11 cycles of 94 °C for 45 s, 65 °C for 45 s, 72 °C for 1.5 min, followed by 26 cycles of 94 °C for 45 s, 55 °C for 45 s, 72 °C for 1.5 min, and a final step of 72 °C for 10 min [35].

### *spa* typing

*spa* typing was performed in a 20 mL reaction including 9.5 mL DDW, 9.5 mL PCR mix (ReddyMix, Tamar Laboratory Supplies, Mevaseret Zion), and primers 1514R, 1113 F (4pmol each). Thermocycling conditions were set at 94 °C for 5 min, followed by 35 cycles of 94 °C for 45 s, 60 °C for 45 s, 72 °C for 90 s, followed by a final extension step of 72 °C for 10 min. Cycle sequencing was carried out using the BigDye Terminator v1.1 chemistry (Applied Biosystems, Foster City, CA, USA) according to manufacturer’s protocol. Cycle sequencing products were purified by gel using Performa DTR according to the manufacturer’s recommendation. Electrophoresis was carried out on a 3730 × l Genetic Analyzer (Applied Biosystems) and the analysis was done using sequencing Analysis v.5.3.1 software (Applied Biosystems). *spa* typing analysis was performed using BioNumerics 7.5 software [36].

### PFGE

Agarose-embedded (Lonza, USA) SA DNA was digested with SmaI (Fermentas, Lithuania) followed by gel electrophoresis in the CHEF MAPPER (Bio-Rad) system. Electrophoresis conditions were 14 °C, 0.5 × Tris-borate-EDTA buffer (Sigma, Switzerland), initial pulse 5 s, final pulse 50 s, 6 V, 18 h. *Salmonella Braenderup* H9812 restricted with XbaI (Fermentas, Lithuania) was used as a marker. PFGE restriction patterns were analyzed by the BioNumerics software (Applied Maths). The pulsotypes were compared using the band-based DICE similarity coefficient with 1% optimization and tolerance. The un-weighted pair group method with arithmetic mean (UPGMA) algorithm was used for cluster analysis [37].

### Statistical analysis

Statistical analysis was performed using SPSS for windows version 21 (IBM Analytics). Proportion tests, Chi-square tests, and Wilcoxon signed ranks test were used for the univariate analysis using α = 0.05 and 95% confidence interval (CI). Statistical significance was based on *p* ≤ 0.05.

## Results

A total of 58/109 students of the 2012 class (53%) participated in our study: 31 students of the 4-year track and 27 students of the 3-year track. There were 30 females and 28 males; the age range was 23–35 years. All 58 students participated during the entire study; three samples were taken from each of these students. Overall, 65 SA isolates were cultured from 32 students in the entire study period: 13 MRSA strains from seven students and 52 MSSA strains from 28 students.

### First sample (pre-clinical sampling)

**Fig. 1:**
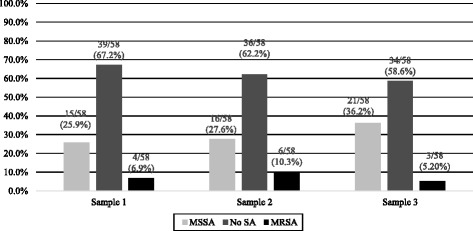
Staphylococcus aureus carriage rates among medical students by sample

**Fig. 2:**
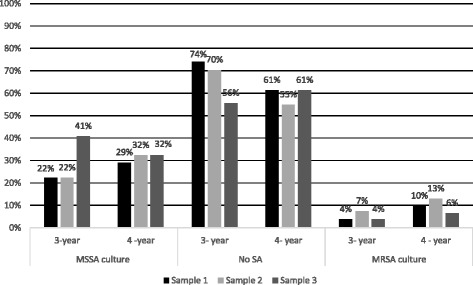
Carriage rates of Staphylococcus aureus isolates according to medical track and sampling time

Overall, 19 (33%) SA isolates were cultured from 7 (12%) students of the three-year track and 12 (20%) students of the 4-year track (Figs. 1 and 2). Four (7%) MRSA isolates were identified: three from students of the 4-year track (10%) and one from a student of the 3-year track (4%). No significant differences in the rates of MSSA and MRSA isolation between the medical tracks were noted (*p =* 0.5; Fig. 2).

### Second sample (first clinical sampling)

Thirteen months after the first pre-clinical samples, 22 SA (38%) isolates were cultured from 30% (eight of 27) of the 3-year track students and 45% (14 of 31) of the 4-year track students (Figs. 1 and 2). Further analysis revealed 16 MSSA (28%) and 6 MRSA isolates (10%) (Fig. 1). No significant differences were noted in the rates of MSSA and MRSA isolation between the medical tracks (*p* = 0.47) or between the first and second samples (*p =* 0.76; Fig. 2).

### Third sample (second clinical sampling)

The last sample took place 6 months following the second sample and included 24 (41%) SA isolates cultured from 44% (12 of 27) of the 3-year track students and 39% (12 of 31) of the 4-year track students (Figs. 1 and 2). Overall, 21 MSSA (36%) and three MRSA isolates (5%) were identified. No significant differences in the rates of MSSA and MRSA isolation between the medical tracks were noted (*p* = 0.74). Compared with the second sample, an increase in the carriage rate of MSSA was noted in students of the 3-year track (*p* = 0.01; Fig. 2). Non-significant increases in the overall SA carriage between the pre-clinical sample and the third sample (*p =* 0.21) and between the second sample and the third sample (*p* = 0.07) were noted.

### Phenotypic and genotypic characterization of MRSA isolates

**Fig. 3:**
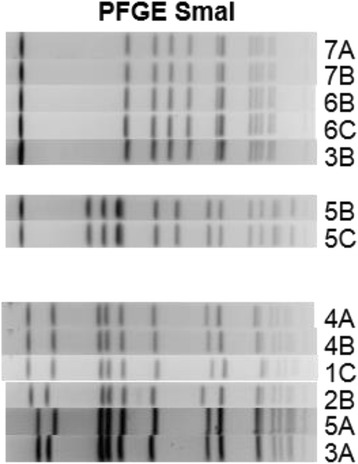
Pulsed-field gel electrophoresis analysis of methicillin-resistant Staphylococcus aureus strains found in the study. * The numbers represent the students with MRSA carriage as mentioned in Fig. 4; the *capital letters* represent the sample time. For example: 1C is a clone cultured from student 1 on the third sample of the study

The *mec*A gene was detected in all MRSA isolates. According to PFGE analysis, three closely-related clones of MRSA strains were identified (Fig. 3, Table 2) [37]. The first clone was isolated exclusively from the 4-year track students; this clone carried SCC*mec* IV, was PVL negative, and typed as *spa* type t032. The second clone was isolated from a single student of the 3-year track and was PVL negative, *spa* type t12435, with an untypable SCC*mec*. The third clone was cultured from students in both medical tracks and carried SCC*mec* II, was PVL negative, and typed as *spa* type t002.

**Table 2:** Summary of molecular characteristics of carried methicillin-resistant *Staphylococcus aureus* strains

	3-year track (*n* = 4)	4-year track (*n* = 9)	Total (*n* = 13)
SCC*mec* II, t002	2	5	7
SCC*mec* IV, t032	0	4	4
t12435^a^	2	0	2

^a^Untypable SCC*mec*

All MRSA Isolates were PVL-negative

Staphylococcal Cassette Chromosome *mec* (SCC*mec*)

Overall, 38% of MRSA isolates (5 out of 13) were resistant to clindamycin whereas only 6% of MSSA isolates were clindamycin resistant (*p* = 0.01; Table 3). Clindamycin resistance rates differed among the various MRSA clones: half of SCC*mec* II, t002 isolates were resistant while none were resistant in the SCC*mec* IV, t032 clone. All MRSA isolates were susceptible to TMP-SMX, tetracycline, rifampicin, fusidic acid, mupirocin, and vancomycin, and were non-MDR.

**Table 3:** Antibiotic resistance rates of Staphylococcus aureus isolates

	3-year program		4-year program		Total	
	MSSA (*n* = 23)	MRSA (*n* = 4)	MSSA (*n* = 29)	MRSA (*n* = 9)	MSSA (*n* = 52)	MRSA (*n* = 13)
Ciprofloxacin	0%	0%	3%	22%	2%	15%
Clindamycin	4%	100%	7%	11%	6%	38%
Erythromycin	60%	25%	21%	22%	38%	23%
Gentamicin	17%	75%	3%	44%	10%	54%

All isolates were susceptible to trimethoprim-sulfamethoxazole (TMP-SMX), fusidic acid, mupirocin, tetracycline, rifampin, and vancomycin

*MSSA* Methicillin-susceptible *Staphylococcus aureus*, *MRSA* Methicillin-resistant *Staphylococcus aureus*

### Carriage of MRSA over multiple sampling times

**Fig. 4:**
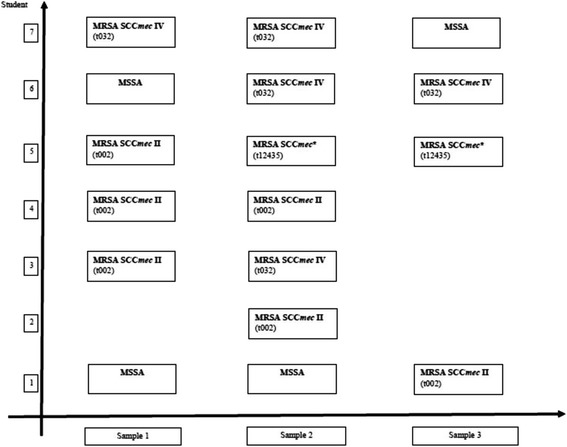
Time-line of methicillin-resistant S. aureus carriage

Five of the seven (71%) students who were colonized with MRSA carried the bacterium in more than one sample (Fig. 4); two students carried different MRSA clones at various sampling times. Four students (57%) had MRSA carriage during the pre-clinical studies. None of the students carried MRSA and MSSA simultaneously.

## Discussion

The carriage of SA increased in medical students along the sampling time (33, 38, and 41%, respectively). A non-significant increase (*p* = 0.07) was noted in the overall rise of SA carriage. However, a statistically significant increase in the MSSA carriage among the 3-year track students was evident when the second sample was compared with the third; both were taken during the clinical stages of studies. Thus, it is possible that a larger sample would also lead to a significant overall rise in SA carriage between the preclinical and clinical parts of the studies. This was indeed observed in three of eight studies comparing preclinical and clinical medical students [9, 23, 26].

In the multiple samplings we performed, up to 10% of the students carried MRSA. This rate is higher than observed in other studies looking at medical students, where carriage rates of 0–5.4% were noted (Table 1). In fact, according to a systematic review of MRSA carriage among healthcare workers in Europe and the United States, it is close to the reported carriage rates of nursing staff [6]. Possible explanations for this difference in rates of MRSA carriage among medical students may be from various sampling times in the studies, MRSA prevalence rates in hospitals and communities in different countries, and the relatively small sample size of studies.

The MRSA strains we isolated are compatible with HA-MRSA strains. We base this observation on the molecular characteristics of the strains and epidemiologic studies from Israel. Both SCC*mec* II, t002 and SCC*mec* IV, t032 are common hospital MRSA strains in Israel [38, 39]. Indeed, SCC*mec* II, t002 is reported to be the second most common clone in Israeli hospitals [38]. Although mostly described in CA-MRSA strains, up to 30% of HA-MRSA isolates in Israel carry SCC*mec* types IV and V [38, 39]. A recent report of community-onset MRSA in Israel demonstrated SCC*mec* IV, t032 to represent nearly 30% of SCC*mec* IV isolates; thus this strain is commonly isolated both in Israeli hospitals and in patients from the community [40]. In contrast, typical CA-MRSA strains in Israel have different genotypes: USA300 (SCC*mec* IV, t008, PVL+) and SCC*mec* IV, t991, PVL- [40]. The third strain in our study, t12435, is relatively rare, being isolated only a few times from hospitalized patients in northern Israel according to data from the Israeli National *Staphylococcus aureus* Reference Center. Although non-MDR phenotype of MRSA is commonly described among CA-MRSA strains, this phenotype was also common among SCC*mec* IV, t032 isolates from Israeli hospitals with relatively low clindamycin resistance rates [1, 2, 40]. Our findings differ from other reports of MRSA in medical students: predominantly CA-MRSA clones (as suggested by the authors) were isolated from medical students in China, Serbia, Canada, Colombia and Taiwan, suggesting these countries have a different MRSA epidemiology [15, 19, 21, 24, 28, 29].

Two additional observations are worth mentioning: we did not find new, non-local MRSA clones among the students, although the 3-year track students arrived from various countries in Europe. This probably implies acquisition of MRSA clones in Israel but can also represent acquisition of global MRSA strains from Europe [41]. The second observation is that although students carried MRSA isolates compatible with HA-MRSA clones, the acquisition of the majority of MRSA isolates, including two of three clones, took place before the clinical part of the studies, namely before the exposure to the healthcare system occurred. Indeed, 80% of community-onset MRSA isolates among patients insured by a large health maintenance organization in Israel were recently shown to be of nosocomial origin [40]. This can explain the acquisition of HA-MRSA clones by students before clinical rotations. Another explanation may be that some students had unrecognized healthcare exposure early in their studies.

In assessing carriage over time, we observed that in two students multiple MRSA clones were carried on different occasions. Compared with MSSA, less is known about the natural history of MRSA carriage and therefore comparison to MSSA data may seem tempting. However, this comparison is problematic as the variety of genotypes and prevalence in the community is larger for MSSA in most geographic locales [1, 3]. Carrying multiple genotypes of MRSA over time may imply acquisition of a new strain from a fellow student or during healthcare exposure. Furthermore, we noticed that only one of seven students colonized with MRSA can be defined as a persistent carrier and even so with multiple genotypes. Lastly, we observed that there was no simultaneous carriage of MRSA and MSSA isolates. Simultaneous carriage of MRSA and MSSA was previously reported either as a rare occurrence, assuming competition for colonization space and nutrients, or in contrast, as a relatively common occurrence [42, 43].

Our study had some limitations. The number of students participating was relatively small and the sampling was not done simultaneously in all students. The nose was the only site tested. Additionally, only one medical school participated. This may limit the power of the study and the generalization for all areas of Israel. The strengths of the study are in the prospective collection of data over multiple times in preclinical and clinical parts of the students’ education, the participation of all students during the whole study, and the genotyping of MRSA strains.

## Conclusion

MSSA carriage rates increased during the clinical part of the studies in medical students in Israel. Higher rates of MRSA carriage were evident in this study compared with previous reports of medical students. MRSA strains carried were genotypically similar to healthcare-associated clones in Israel; however, MRSA carriage occurred in the majority of students before healthcare exposure, probably representing community-acquisition of hospital strains. Until official recommendations regarding screening and interventions for MRSA in medical students will be published, a call for good personal hygiene is in place.

## Abbreviations

CA-MRSA: Community-associated methicillin-resistant *Staphylococcus aureus*
HA-MRSA: Healthcare-associated methicillin-resistant *Staphylococcus aureus*
MRSA: Methicillin-resistant *Staphylococcus aureus*
PVL: Panton-Valentine Leukocidin
SA: *Staphylococcus aureus*
